# Supplementary material for: Niacin ameliorates ulcerative colitis via prostaglandin D2‐mediated D prostanoid receptor 1 activation
Source: EMBO Mol Med. 2017 Mar 27;9(5):571–88. doi: 10.15252/emmm.201606987 (PMC5412792; doi:10.15252/emmm.201606987)
Supplement: Supplementary file 1 — Appendix [file EMMM-9-571-s001.pdf]

Table of contents

Appendix Table S1. Demographics and baseline characteristics of patients.

Appendix Table S2. Subscores of Mayo score of recruited patients.

Appendix Table S3. Serious adverse effects of niacin treatment.

Appendix Table S4. Complete blood count and routine blood biochemical assays of recruited patients.

Appendix Table S5. Primers for mouse genotyping and RT-PCRs.

Appendix Table S6. Actual P-values indicated as “significant” in the figures.

Appendix Figure S1. Effect of niacin on colonic and urinary 8-isoprostane $PGF_{2\alpha}$ production and serum lipid profile in mice after DSS challenge.

Appendix Figure S2. Urinary levels of PG metabolites and 8-isoprostane $PGF_{2\alpha}$ in UC patients after niacin treatment.

Appendix Figure Legends.

Appendix Tables

Table S1

Demographics and baseline characteristics of patients

	Age (Y)	BMI	Gender	Duration of disease(Y)	Disease extent	Mayo score (0-12)	CRP (mg/L)
1	45	26.0	M	3	left side	6	1
2	43	20.3	F	4	left side	10	7.12
3	47	21.3	M	10	left side	8	74.7
4	56	15.6	F	1.5	left side	10	6
5	31	25.8	M	1.5	left side	9	4
6	43	19.3	F	1	left side	8	4
7	46	23.1	M	2	left side	6	1
8	56	21.3	M	4.5	left side	10	5
9	19	22.9	M	2	left side	8	5
10	60	21.9	M	1	left side	9	1
11	44	18.5	F	6	left side	9	15
12	19	18.4	F	1	left side	10	4
13	39	23.5	M	3	left side	10	1.7
14	45	23.0	F	9	left side	10	3
15	29	17.0	F	1	left side	9	47
16	18	23.7	M	1.5	left side	10	2.1
17	44	22.0	F	1	left side	7	1.9
18	43	21.3	M	5	left side	9	70.8
19	29	17.3	M	1	left side	9	3
20	36	19.5	F	13	left side	10	1.42
21	42	22.2	F	9	left side	9	3
22	21	18.4	M	1.5	left side	8	1
23	43	23.5	M	1	left side	6	2.56
24	35	18.5	M	1	left side	7	0.21
25	54	16.7	M	4	left side	8	11
26	30	20.5	M	1	left side	7	1

Note: All the patients had received both oral conventional regular medicines and consecutive regular retention enema medicines before the study. The oral conventional regular medicines: oral 5- aminosalicylate (5-ASA, eg. 4.0g of Pentasa per day) and corticosteroids (eg. 30-40 mg of oral prednisolone or the equivalent per day). Consecutive regular retention enema treatment: 5mg of dexamethasone, 0.5g of metronidazole, and 5.0g of starch in 100ml saline per day.

Table S2

Subscores of Mayo score of recruited patients

Subscore	Initial	Before niacin treatment	After niacin treatment
Stool frequency	2.00 ± 0.14	2.04 ± 0.16	0.35 ± 0.11**
Rectal bleeding	2.27 ± 0.12	2.08 ± 0.12	0.15 ± 0.11**
Endoscopic findings	2.29 ± 0.10	2.39 ± 0.10	0.46 ± 0.16**
Physician's global assessment	2.10 ± 0.10	2.04 ± 0.09	0.58 ± 0.15**
Mayo score	8.65 ± 0.30	8.54 ± 0.27	1.54 ± 0.45**

**, p<0.01, vs Before niacin treatment; n=26

Table S3

Serious adverse effects of niacin treatment

Serious adverse effect	Incidence rate
Flushing and urticaria	0/26
Dizzy and headache	0/26
Nausea, vomiting, stomach pain	0/26
Hyperglycemia	0/26
Hyperuricemia	0/26
Arrhythmia	0/26
Hepatotoxicity	0/26

Table S4

Complete blood count and routine blood biochemical assays of recruited patients

Blood tests	Before niacin treatment	After niacin treatment
Albumin	34.83 ± 1.15	38.00 ± 1.06*
White blood cell	7.27 ± 0.47 (× 10^9)	6.62 ± 0.41 (× 10^9)
Neutrophil	61.44% ± 2.58%	57.54% ± 1.89%
Hemoglobin	124.2 ± 4.60	133.7± 4.59**
TG	0.98 ± 0.15	1.00 ± 0.10
TC	3.89 ± 0.20	4.30 ± 0.24
HDL	1.27 ± 0.08	1.41 ± 0.15
LDL	2.34 ± 0.21	2.58 ± 0.18

*, $p<0.05$, **, $p<0.01$, vs Before niacin treatment; Albumin, White blood cell, White blood cell, White blood cell, n=24; TG, TC, HDL, LDL, n=10.

Table S5

Primers for mouse genotyping and RT-PCRs

Gene	Sense	Anti-sense
$DP1^{-/-}$	TTCCTTTCCCCCACTGCTCCTAC	CACCACTTAACTAAAGGGC
$DP1^{+/+}$	GCTTAATGCCCCAGGAGTGTGAA	CACCACTTAACTAAAGGGC
$DP2^{-/-}$	TTCCATCCGAGTACGTGCTC	GTGCTCGACGTTGTCACTGA
$DP2^{+/+}$	CGTAGCCGTGAGCCTGCGACTG	GGCGATTGCGGAGCCCACCACT
$DP1^{flox}$	GCTTAATGCCCCAGGAGTGTGAA	CCTACCGGTGGATGTGGAATGTG
$Tie2^{Cre}$	CGCATAACCAGTGAAACAGCATTGC	GTAAACAAGAGCGAGTGGACCATG
$Sm22^{Cre}$	CTCCTTCCAGTCCACAAACGACC	GGCGATCCCTGAACATGTCC
$pVillin^{Cre}$	ATCAACGTTTTCTTTTCGG	ATTTGCCTGCATTACCGGTC
$LysM^{Cre}$	AGGTGTAGAGAAGGCACTCAGC	CTA ATCGCCATCTTCCAGCAGG
DP1	AACCTCTATGACATGCACAGGCG	AAGGCTTGGAGGTCTTCTGAGTC
IL-4	GATCCCCGGGCAGAGC	TGTCGCATCCGTGGATATGG
IL-5	AGCAATGAGACGATGAGGCT	GTACCCCCACGGACAGTTTG
IL-6	TAGTCCTTCCTACCCCAATTTCC	TTGGTCCTTAGCCACTCCTTC
IL-10	GCTCTTACTGACTGGCATGAG	CGCAGCTCTAGGAGCATGTG
IL-1β	GCAACTGTTCCTGAACTCAACT	ATCTTTTGGGGTCCGTCAACT
TNFα	ATGGCCTCCCTCTCATCAGT	TTTGCTACGACGTGGGCTAC
MCP-1	TTAAAAACCTGGATCGGAACCAA	GCATTAGCTTCAGATTTACGGGT
COX1	GATTGTACTCGCACGGGCTAC	GGATAAGGTTGGACCGCACT
COX-2	GCTCACGAAGGAACTCAG	GGATTGGAACAGCAAGGAT
lPGDS	TGCAGCCCAACTTTCAACAAG	TGGTCTCACACTGGTTTTTCCT
hPGDS	GGAAGAGCCGAAATTATTCGCT	ACCACTGCATCAGCTTGACAT
$cPLA_2$	ACCTGCGAGTGTGACAAGAG	CTTGTTGGGGTAGTGGGCAT
β-actin	GGCTGTATTCCCCTCCATCG	CCAGTTGGTAACAATGCCATGT

Table S6

Actual P-values indicated as "significant" in the figures

Figure	Group A	Group B	p-value	Indication
1A	Vehicle	Niacin 300mg/Kg	0.0378	#
1A	Vehicle	Niacin 600mg/Kg	0.0007	##
1B	Vehicle	Niacin 300mg/Kg	0.0411	#
1B	Vehicle	Niacin 600mg/Kg	0.0004	##
1C	Vehicle	Niacin	0.0364	#
1D	Vehicle	Niacin	0.0324	#
1E	Vehicle	Niacin	0.0108	#
2B	WT, Day 5	$DP^{-/-}$, Day 5	< 0.0001	**
2B	WT, Day 6	$DP^{-/-}$, Day 6	< 0.0001	**
2C	WT, Day 4	$DP^{-/-}$, Day 4	0.0095	**
2C	WT, Day 5	$DP^{-/-}$, Day 5	0.0001	**
2C	WT, Day 6	$DP^{-/-}$, Day 6	0.0003	**
2D	WT	$DP^{-/-}$	0.0011	**
2F	WT	$DP^{-/-}$	0.0459	*
3B	WT, Vehicle, Day 7	WT, Niacin, Day 7	0.0445	#
3B	WT, Vehicle, Day 8	WT, Niacin, Day 8	0.0095	##
3B	WT, Vehicle, Day 9	WT, Niacin, Day 9	0.0031	##
3C	WT, Vehicle, Day 6	WT, Niacin, Day 6	0.0204	#
3C	WT, Vehicle, Day 9	WT, Niacin, Day 9	0.0001	##
3E	WT, Vehicle	WT, Niacin	< 0.0001	##
3F	WT, Vehicle	WT, Niacin	0.0205	#
3F	WT, Vehicle	$DP^{-/-}$, Vehicle	0.0012	**
3F	WT, Niacin	$DP^{-/-}$, Niacin	< 0.0001	**
4C	$DP1^{F/F}$	$DP1^{F/F}/Tie2^{Cre}$	0.0015	*
4C	$DP1^{F/F}$	$DP1^{F/F}/Villin^{Cre}$	< 0.0001	*
4C	$DP1^{F/F}$	$DP1^{F/F}/LysM^{Cre}$	< 0.0001	*
4C	$DP1^{F/F}$	$DP1^{F/F}/SM22^{Cre}$	0.0012	*
4D	$DP1^{F/F}$, $DP1^{F/F}/Tie2^{Cre}$, $DP1^{F/F}/Villin^{Cre}$, $DP1^{F/F}/LysM^{Cre}$, $DP1^{F/F}/SM22^{Cre}$		0.0085	**
4E	$DP1^{F/F}$, $DP1^{F/F}/Tie2^{Cre}$, $DP1^{F/F}/Villin^{Cre}$, $DP1^{F/F}/LysM^{Cre}$, $DP1^{F/F}/SM22^{Cre}$		< 0.0001	**
4F	$DP1^{F/F}$	$DP1^{F/F}/Tie2^{Cre}$	0.0038	**
4F	$DP1^{F/F}$	$DP1^{F/F}/Villin^{Cre}$	0.0005	**
4F	$DP1^{F/F}$	$DP1^{F/F}/LysM^{Cre}$	0.0055	**
5C	$DP1^{F/F}$, Vehicle	$DP1^{F/F}$, BW245C	< 0.0001	##
5C	$DP1^{F/F}$, Vehicle	$DP1^{F/F}/Tie2^{Cre}$, Vehicle	0.0005	**

5C	$DP1^{F/F}$, BW245C	$DP1^{F/F}/Tie2^{Cre}$, BW245C	0.0002	**
5D	$DP1^{F/F}$	$DP1^{F/F}/Tie2^{Cre}$	0.0002	**
5E	WT, Vehicle, Day 6	$DP^{-/-}$, Vehicle, Day 6	< 0.0001	**
5E	WT, Vehicle, Day 9	WT, Niacin, Day 9	0.0088	##
5E	WT, Niacin, Day 6	$DP^{-/-}$, Niacin, Day 6	< 0.0001	**
6B	$DP1^{F/F}$	$DP1^{F/F}/Villin^{Cre}$	0.0062	**
6C	$DP1^{F/F}$	$DP1^{F/F}/Villin^{Cre}$	0.0148	*
6D	WT, Vehicle	WT, IL-13	0.0124	#
6D	$DP^{-/-}$, Vehicle	$DP^{-/-}$, IL-13	0.0002	##
6D	WT, IL-13	$DP^{-/-}$, IL-13	< 0.0001	**
6E	WT, Vehicle	WT, Niacin	0.0018	##
6E	WT, Vehicle	$DP^{-/-}$, Vehicle	0.0011	**
6E	WT, Niacin	$DP^{-/-}$, Niacin	0.0001	**
6F	WT, Vehicle	WT, Niacin	< 0.0001	##
6F	WT, Vehicle	$DP^{-/-}$, Vehicle	0.0008	**
6F	WT, Niacin	$DP^{-/-}$, Niacin	< 0.0001	**
7A	$DP1^{F/F}$	$DP1^{F/F}/LysM^{Cre}$	< 0.0001	**
7B	$DP1^{F/F}$	$DP1^{F/F}/LysM^{Cre}$	0.0079	**
7D	$DP1^{F/F}$, TNF-a	$DP1^{F/F}/LysM^{Cre}$, TNF-a	0.0241	*
7D	$DP1^{F/F}$, MCP-1	$DP1^{F/F}/LysM^{Cre}$, MCP-1	0.0104	*
7D	$DP1^{F/F}$, IL-5	$DP1^{F/F}/LysM^{Cre}$, IL-5	0.0371	*
7D	$DP1^{F/F}$, IL-10	$DP1^{F/F}/LysM^{Cre}$, IL-10	0.0465	*
7E	WT, Vehicle	WT, Niacin	0.0004	##
7E	WT, Vehicle	$DP^{-/-}$, Vehicle	< 0.0001	**
7E	WT, Niacin	$DP^{-/-}$, Niacin	< 0.0001	**
7F	WT, Vehicle, MCP1	WT, Niacin, MCP1	0.0112	#
7F	WT, Niacin, MCP1	$DP^{-/-}$, Niacin, MCP-1	0.0097	**
7F	WT, Vehicle, IL-5	WT, Niacin, IL-5	0.0461	#
7F	WT, Niacin, IL-5	$DP^{-/-}$, Niacin, IL-5	0.0268	*
7F	WT, Vehicle, IL-10	WT, Niacin, IL-10	0.0424	#
7F	WT, Vehicle, IL-10	$DP^{-/-}$, Vehicle, IL-10	0.0232	*
7F	WT, Niacin, IL-10	$DP^{-/-}$, Niacin, IL-10	0.0173	*
8B	Niacin 0h	Niacin 4h	0.0105	*
8D	initial	Niacin treatment	< 0.0001	**
8D	No response	Niacin treatment	< 0.0001	**
EV1A	Vehicle	Niacin	0.0081	##
EV1B	Vehicle, PGEM	Niacin, PGEM	0.0022	##
EV1B	Vehicle, PGIM	Niacin, PGIM	0.0049	##
EV1B	Vehicle, PGFM	Niacin, PGFM	0.0493	#
EV2B	WT, Day 3	$DP1^{-/-}$, Day 3	0.0062	**
EV2B	WT, Day 4	$DP1^{-/-}$, Day 4	0.0004	**
EV2B	WT, Day 5	$DP1^{-/-}$, Day 5	0.0009	**
EV2B	WT, Day 6	$DP1^{-/-}$, Day 6	0.0014	**

EV2C	WT, Day 4	$DP1^{-/-}$, Day 4	0.0323	*
EV2C	WT, Day 5	$DP1^{-/-}$, Day 5	0.0045	**
EV2C	WT, Day 6	$DP1^{-/-}$, Day 6	0.0004	**
EV2D	WT	$DP1^{-/-}$	0.0087	**
EV3A	WT, Vehicle, Day 6	WT, Niacin, Day 6	0.0143	#
EV3A	WT, Vehicle, Day 4	$DP1^{-/-}$, Vehicle, Day 4	0.0208	*
EV3A	WT, Niacin, Day 4	$DP1^{-/-}$, Niacin, Day 4	0.0382	*
EV3A	WT, Vehicle, Day 5	$DP1^{-/-}$, Vehicle, Day 5	0.0020	**
EV3A	WT, Niacin, Day 5	$DP1^{-/-}$, Niacin, Day 5	< 0.0001	**
EV3A	WT, Vehicle, Day 6	$DP1^{-/-}$, Vehicle, Day 6	0.0013	**
EV3A	WT, Niacin, Day 6	$DP1^{-/-}$, Niacin, Day 6	< 0.0001	**
EV3B	WT, Vehicle, Day 3	$DP1^{-/-}$, Vehicle, Day 3	0.0060	**
EV3B	WT, Niacin, Day 3	$DP1^{-/-}$, Niacin, Day 3	0.0047	**
EV3B	WT, Vehicle, Day 4	$DP1^{-/-}$, Vehicle, Day 4	0.0132	*
EV3B	WT, Niacin, Day 4	$DP1^{-/-}$, Niacin, Day 4	0.0035	**
EV3B	WT, Vehicle, Day 5	$DP1^{-/-}$, Vehicle, Day 5	0.0075	**
EV3B	WT, Niacin, Day 5	$DP1^{-/-}$, Niacin, Day 5	0.0008	**
EV3B	WT, Vehicle, Day 6	$DP1^{-/-}$, Vehicle, Day 6	0.0063	**
EV3B	WT, Niacin, Day 6	$DP1^{-/-}$, Niacin, Day 6	0.0010	**
EV3C	WT, Vehicle	WT, Niacin	0.0041	##
EV3C	WT, Vehicle	$DP1^{-/-}$, Vehicle	< 0.0001	**
EV3C	WT, Niacin	$DP1^{-/-}$, Niacin	< 0.0001	**
EV4A	$DP1^{F/F}$, $DP1^{F/F}/Tie2^{Cre}$, $DP1^{F/F}/Villin^{Cre}$, $DP1^{F/F}/LysM^{Cre}$, $DP1^{F/F}/SM22^{Cre}$ Body weight loss		0.0296	*
EV4A	$DP1^{F/F}$, $DP1^{F/F}/Tie2^{Cre}$, $DP1^{F/F}/Villin^{Cre}$, $DP1^{F/F}/LysM^{Cre}$, $DP1^{F/F}/SM22^{Cre}$ DAI		0.0431	*
EV4A	$DP1^{F/F}$, colon length	$DP1^{F/F}/Tie2^{Cre}$, colon length	0.0009	**
EV4A	$DP1^{F/F}$, colon length	$DP1^{F/F}/Villin^{Cre}$, colon length	0.0014	**
EV4A	$DP1^{F/F}$, colon length	$DP1^{F/F}/LysM^{Cre}$, colon length	0.0012	**
EV4B	$DP1^{F/F}$	$DP1^{F/F}/Tie2^{Cre}$	< 0.0001	**
EV4C	$DP1^{F/F}$	$DP1^{F/F}/Villin^{Cre}$	< 0.0001	**
EV4D	$DP1^{F/F}$	$DP1^{F/F}/LysM^{Cre}$	0.0002	**
EV5A	WT, Vehicle, Day 8	WT, PGD_2, Day 8	0.0146	#
EV5A	WT, Vehicle, Day 9	WT, PGD_2, Day 9	0.0006	##
EV5B	WT, Vehicle, Day 9	WT, PGD_2, Day 9	0.0106	#
EV5C	WT, Vehicle	WT, PGD_2	0.0012	##
EV5D	WT, Vehicle	WT, PGD_2	0.0001	##
EV5E	WT, Vehicle	WT, PGD_2	0.0101	#
EV5F	WT, Vehicle	WT, PGD_2	0.0002	##
Appendix Fig S1B	Vehicle, TC	Niacin, TC	0.0088	##
Appendix	Vehicle, TG	Niacin, TG	0.0319	#

**Fig S1B**				
**Appendix Fig S1B**	**Vehicle, LDL**	**Niacin, LDL**	**0.0015**	**##**
**Appendix Fig S2A**	**Niacin 0h**	**Niacin 4h**	**0.0216**	*****
**Appendix Table S2**	**Before niacin treatment, Stool frequency**	**After niacin treatment, Stool frequency**	**< 0.0001**	******
**Appendix Table S2**	**Before niacin treatment, Rectal bleeding**	**After niacin treatment, Rectal bleeding**	**< 0.0001**	******
**Appendix Table S2**	**Before niacin treatment, Endoscopic findings**	**After niacin treatment,** Endoscopic findings	**< 0.0001**	******
**Appendix Table S2**	**Before niacin treatment, Physician's global assessment**	**After niacin treatment, Physician's global assessment**	**< 0.0001**	******
**Appendix Table S2**	**Before niacin treatment, Mayo score**	**Before niacin treatment, Mayo score**	**< 0.0001**	******
**Appendix Table S4**	**Before niacin treatment, Albumin**	**After niacin treatment, Albumin**	**0.0108**	*****
**Appendix Table S4**	**Before niacin treatment, Hemoglobin**	**Before niacin treatment, Hemoglobin**	**0.0059**	******

Appendix Figure S1

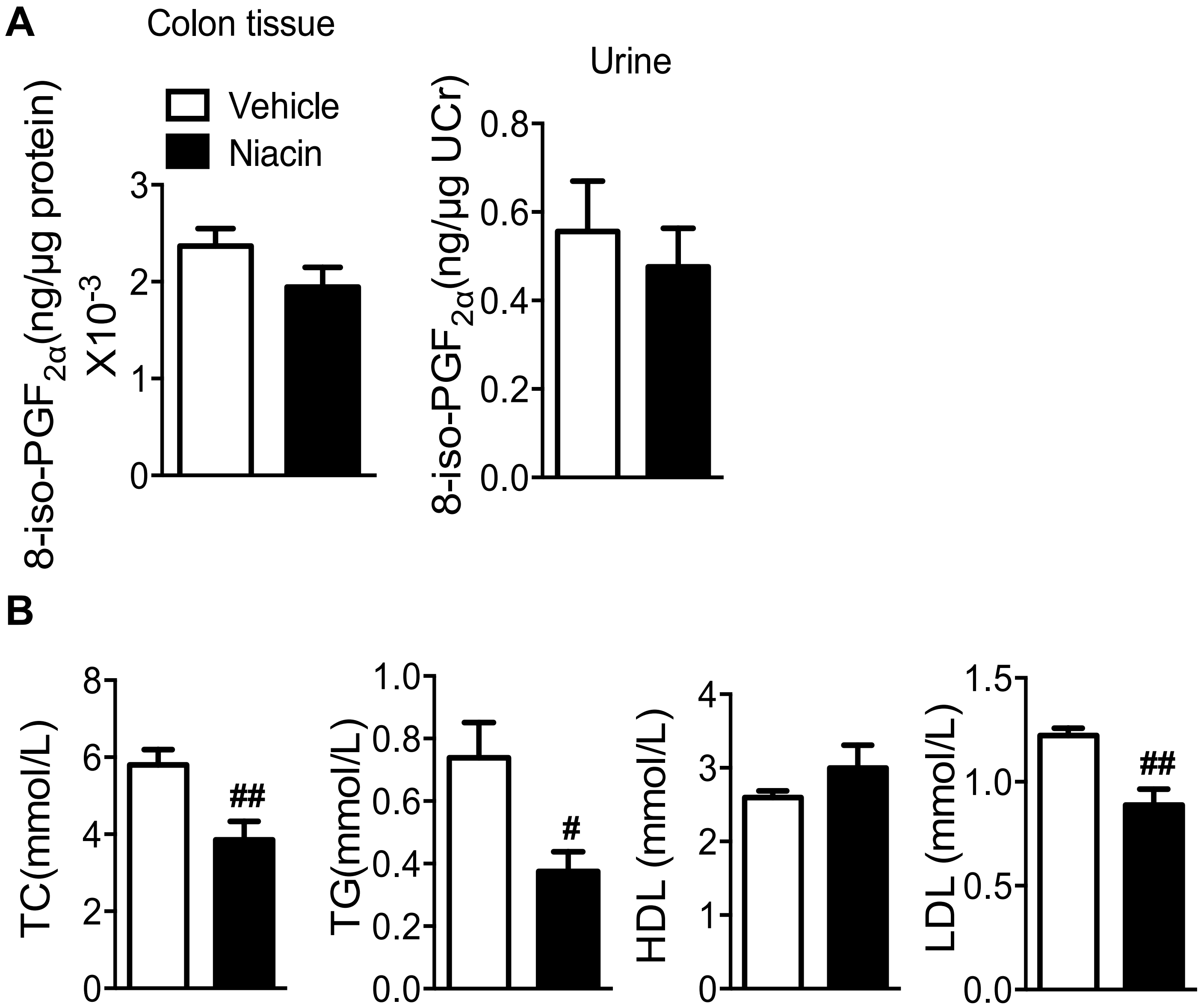

Appendix Figure S2

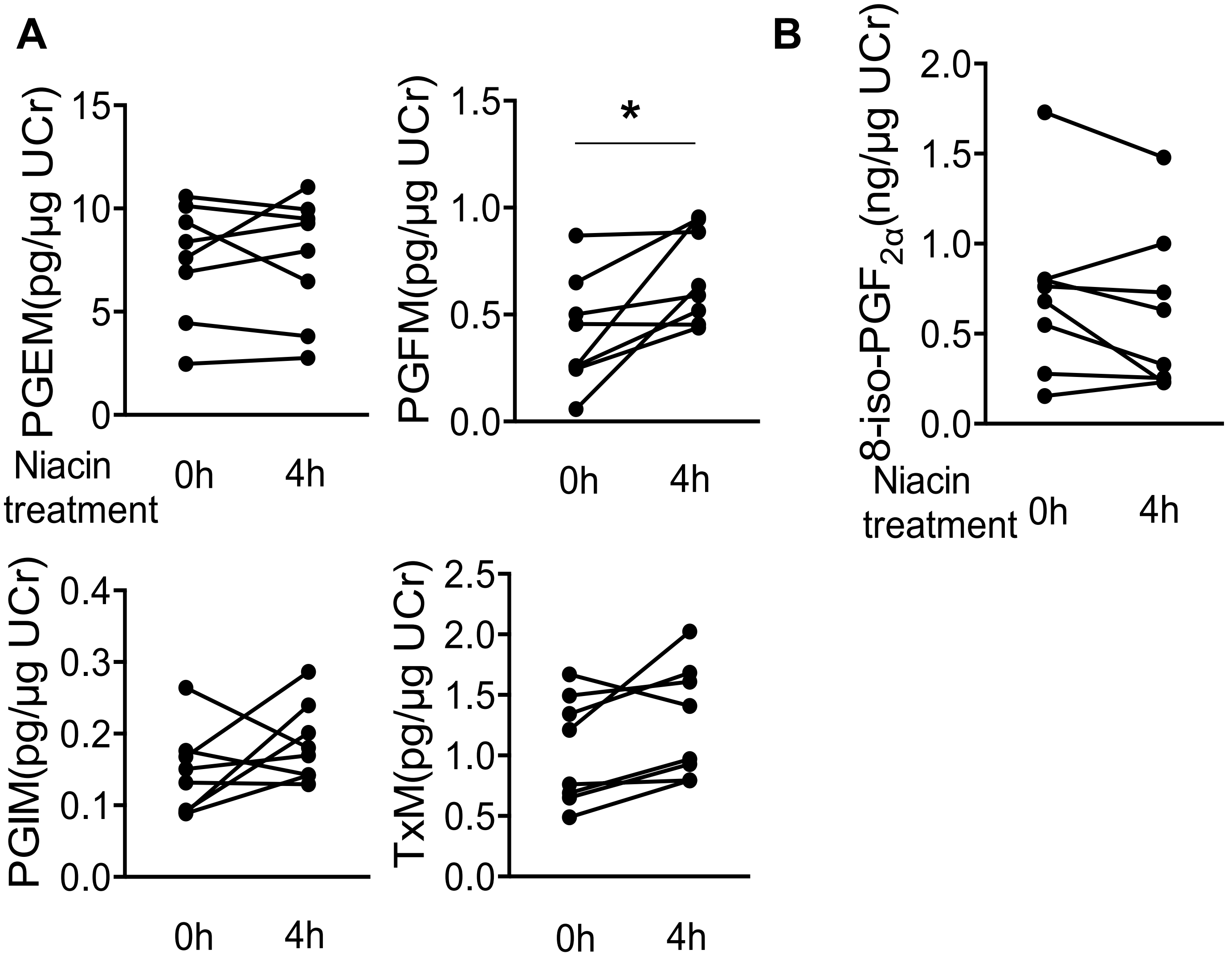

Appendix figure legends

Figure S1

Effect of niacin on colonic and urinary 8-isoprostane $PGF_{2\alpha}$ production and serum lipid profile in mice after DSS challenge.

(A) Mass spectrometry analysis of 8-isoprostane $PGF_{2\alpha}$ levels in colon tissues and urine from DSS-challenged mice. n=7.

(B) Measurement of serum TG, TC, LDL and HDL in mice after DSS challenge. vehicle, n=7; niacin, n=6.

Data information: Data are shown as mean ± SEM. Statistical significance was determined using unpaired Student's t tests. *, $p<0.05$, **, $p<0.01$.

Figure S2

Urinary levels of PG metabolites and 8-isoprostane $PGF_{2\alpha}$ **in UC patients after niacin treatment.**

(A) Effect of niacin retention enema on urinary PGEM, PGFM, PGIM and TxM production in patients.

(B) Effect of niacin retention enema on 8-isoprostane $PGF_{2\alpha}$ production in urine from patients.

Data information: P-values were calculated using paired Student's t-test. *, $p<0.05$ as indicated, (A-B) n=8**.**
