# Supplementary material for: Niacin ameliorates ulcerative colitis via prostaglandin D2‐mediated D prostanoid receptor 1 activation
Source: EMBO Mol Med. 2017 Mar 27;9(5):571–88. doi: 10.15252/emmm.201606987 (PMC5412792; doi:10.15252/emmm.201606987)
Supplement: Supplementary file 2 — Expanded View Figures PDF [file EMMM-9-571-s002.pdf]

Expanded View Figures

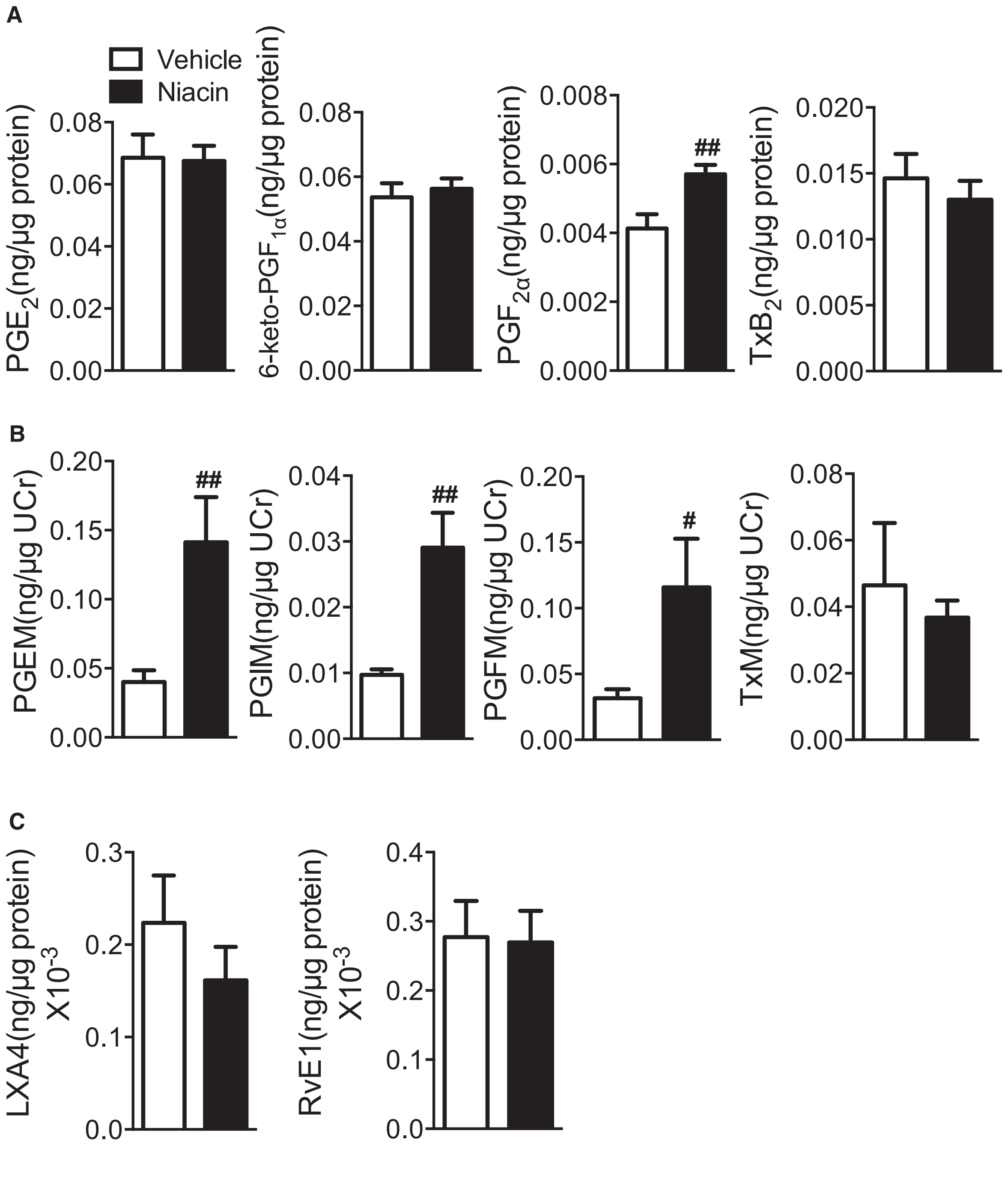

Figure EV1. Effect of niacin on PG, RvE1, and LXA4 production in mice after DSS challenge.

A Mass spectrometry analysis of PG production in colons from niacin (600 mg/kg)-treated mice after DSS challenge. Vehicle, n = 6; niacin 600 mg/kg, n = 7.

B Measurement of urinary metabolite of PGs in niacin (600 mg/kg)-treated mice. PGEM, 11α-hydroxy-9,15-dioxo-2,3,4,5-tetranor-prostane-1,20-dioic acid; PGIM, 2,3-dinor-6-keto-$PGF_{1\alpha}$; TxM, 2,3-dinor-TxB_2; PGFM, 13,14-dihydro-15-keto-$PGF_{2\alpha}$. n = 6.

C Mass spectrometry analysis of RvE1 and LXA4 production in colons from niacin (600 mg/kg)-treated mice after DSS challenge. n = 6.

Data information: Data are shown as mean ± SEM. Data are representative of at least two independent experiments. Statistical significance was determined using unpaired Student's t-tests. (A) $^{\#\#}P < 0.01$. (B) $^{\#}P < 0.05$, $^{\#\#}P < 0.01$.

© 2017 The Authors

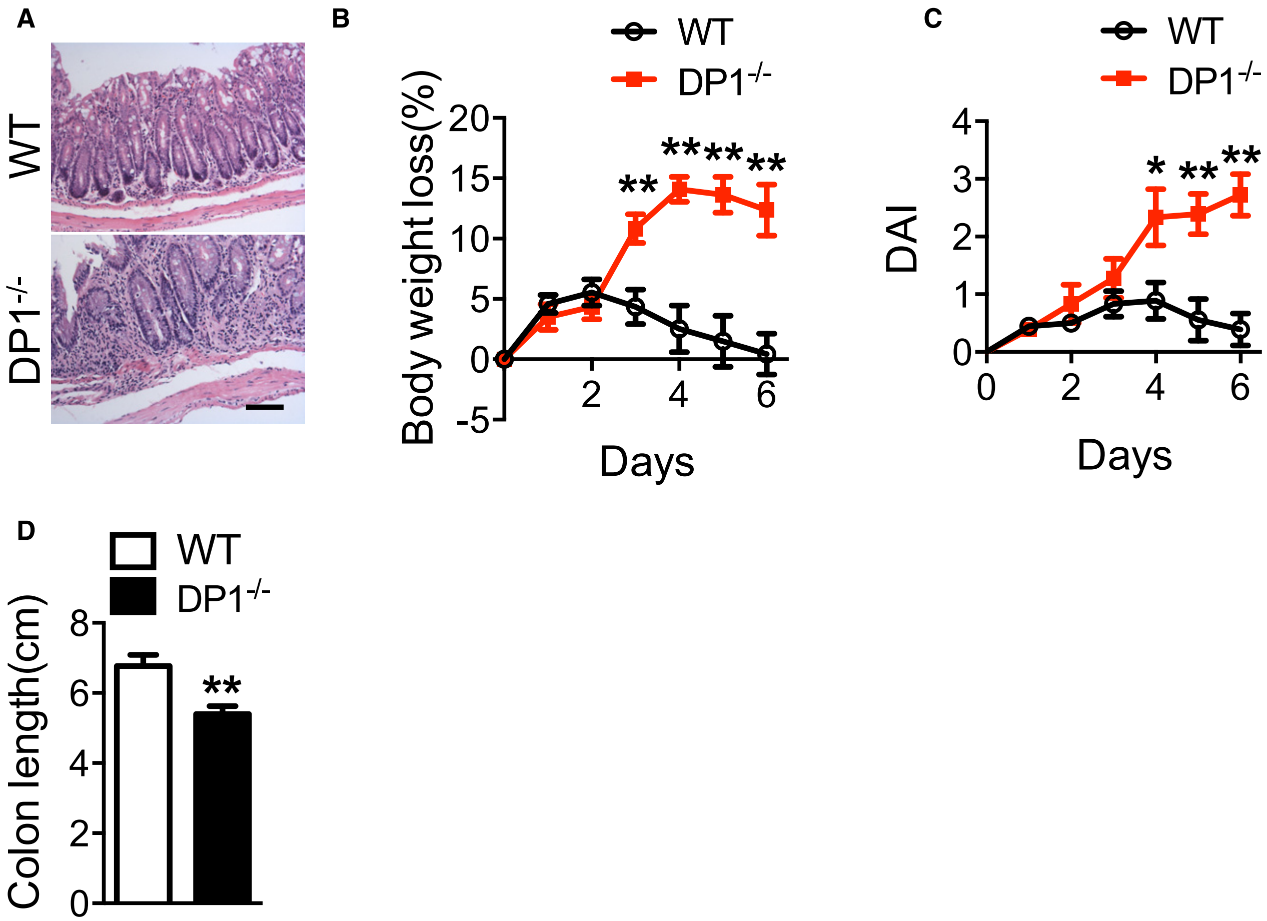

Figure EV2. DP1 deficiency augments TNBS-induced colitis in mice.

A H&E staining of histological sections in the distal colon from the mice after TNBS challenge. Scale bar: 100 μm. Graphs represent overall histology score.

B–D Body weight loss (B), disease activity index (DAI, C), and colon length (D) of $DP1^{-/-}$ and WT mice in response to 2.5% TNBS challenge.

Data information: Data are shown as mean ± SEM. Statistical significance was determined using unpaired Student's *t*-tests. $^{*}P < 0.05$, $^{**}P < 0.01$ compared with WT; $n = 6$.

 © 2017 The Authors

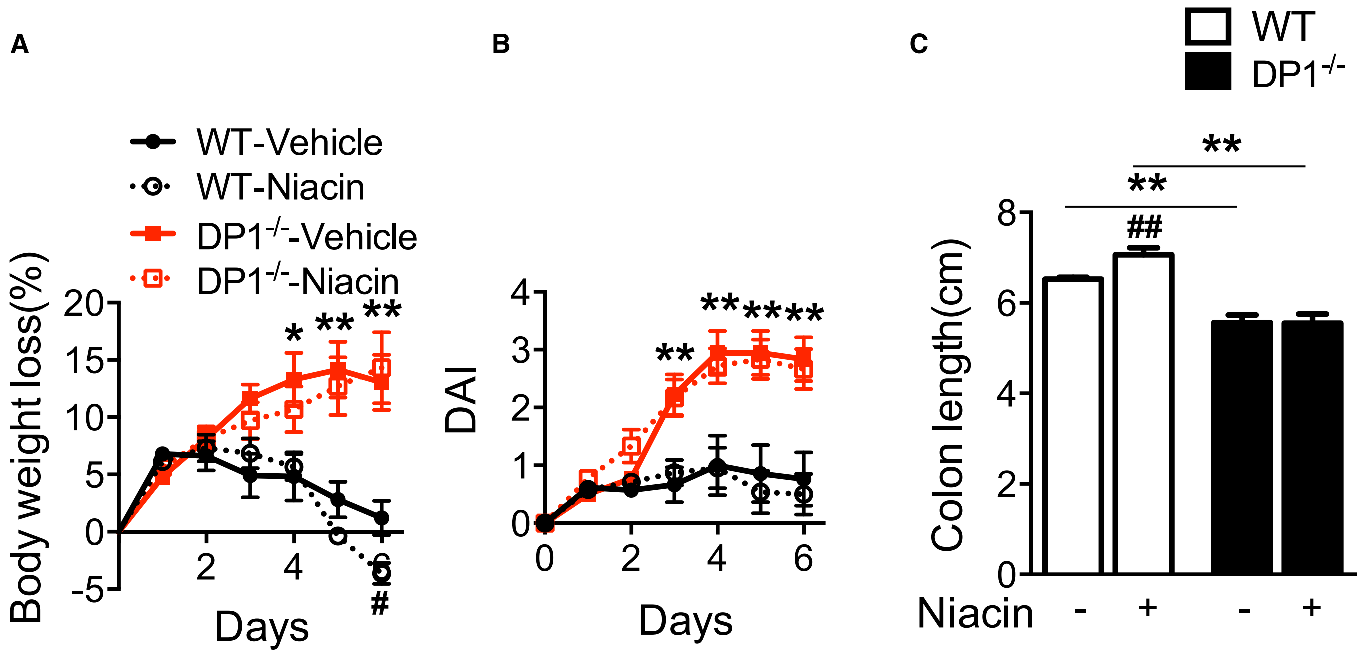

Figure EV3. Niacin protects mice against from TNBS-induced colitis.

A–C Effect of niacin treatment on body weight loss (A), disease activity index (DAI, B), and colon length (centimeter) (C) of DP1$^{-/-}$ and WT mice in response to 2.5% TNBS challenge.

Data information: Data are shown as mean ± SEM. Statistical significance was determined using two-way ANOVA followed by a Bonferroni *post hoc* test. $^{\#}P < 0.05$, $^{\#\#}P < 0.01$ vs. vehicle. $^{*}P < 0.05$, $^{**}P < 0.01$ compared with WT. WT-vehicle, $n = 7$; WT-niacin, $n = 8$; DP1$^{-/-}$-vehicle, DP1$^{-/-}$-niacin, $n = 6$.

© 2017 The Authors

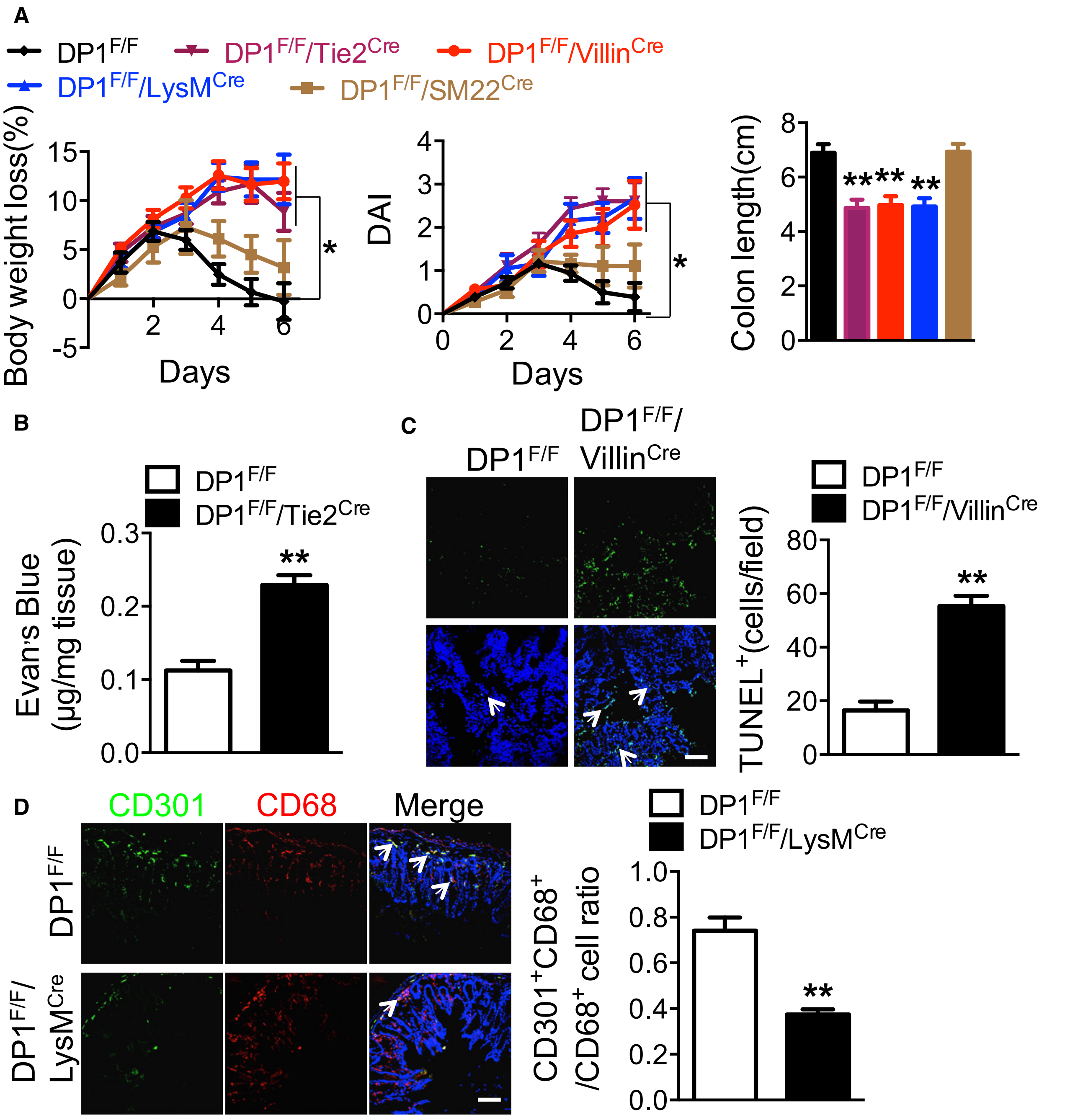

Figure EV4. TNBS-induced colitis in cell-specific DP1-deficient mice.

A Body weight loss, disease activity index (DAI), and colon length of DP1$^{F/F}$/Tie2^{Cre} mice, DP1$^{F/F}$/Villin^{Cre} mice, DP1$^{F/F}$/LysM^{Cre} mice, and DP1$^{F/F}$/SM22^{Cre} mice in response to TNBS treatment.

B Quantitative measurement of vascular permeability by dye leakage in the colonic mucosa from TNBS-challenged DP1$^{F/F}$/Tie2^{Cre} and DP1$^{F/F}$ mice. The mice were sacrificed at day 6.

C TUNEL assay (left) and quantitation (right) in colonic tissues from TNBS-challenged DP1$^{F/F}$/Villin^{Cre} and DP1$^{F/F}$ mice. The arrows indicate the TUNEL^{+} cells (left). Scale bar: 100 µm. DP1$^{F/F}$, $n = 7$; DP1$^{F/F}$/Villin^{Cre}, $n = 8$.

D Representative immunofluorescent staining (left) and quantitation (right) of CD301^{+}CD68^{+} cells in colonic tissues from TNBS-challenged DP1$^{F/F}$/LysM^{Cre} and DP1$^{F/F}$ mice. The arrows indicate the CD301^{+}CD68^{+} cells (left). Scale bar: 100 µm. DP1$^{F/F}$, $n = 8$; DP1$^{F/F}$/LysM^{Cre}, $n = 6$.

Data information: Data are shown as mean ± SEM. Statistical significance was determined using two-way ANOVA followed by a Bonferroni *post hoc* test (A) and unpaired Student's *t*-tests (B–D). (A, B) *$P < 0.05$, **$P < 0.01$ compared with DP1$^{F/F}$. DP1$^{F/F}$, DP1$^{F/F}$/Tie2^{Cre}, DP1$^{F/F}$/LysM^{Cre}, DP1$^{F/F}$/SM22^{Cre} $n = 6$; DP1$^{F/F}$/Villin^{Cre}, $n = 7$. (C, D) **$P < 0.01$ vs. DP1$^{F/F}$.

© 2017 The Authors

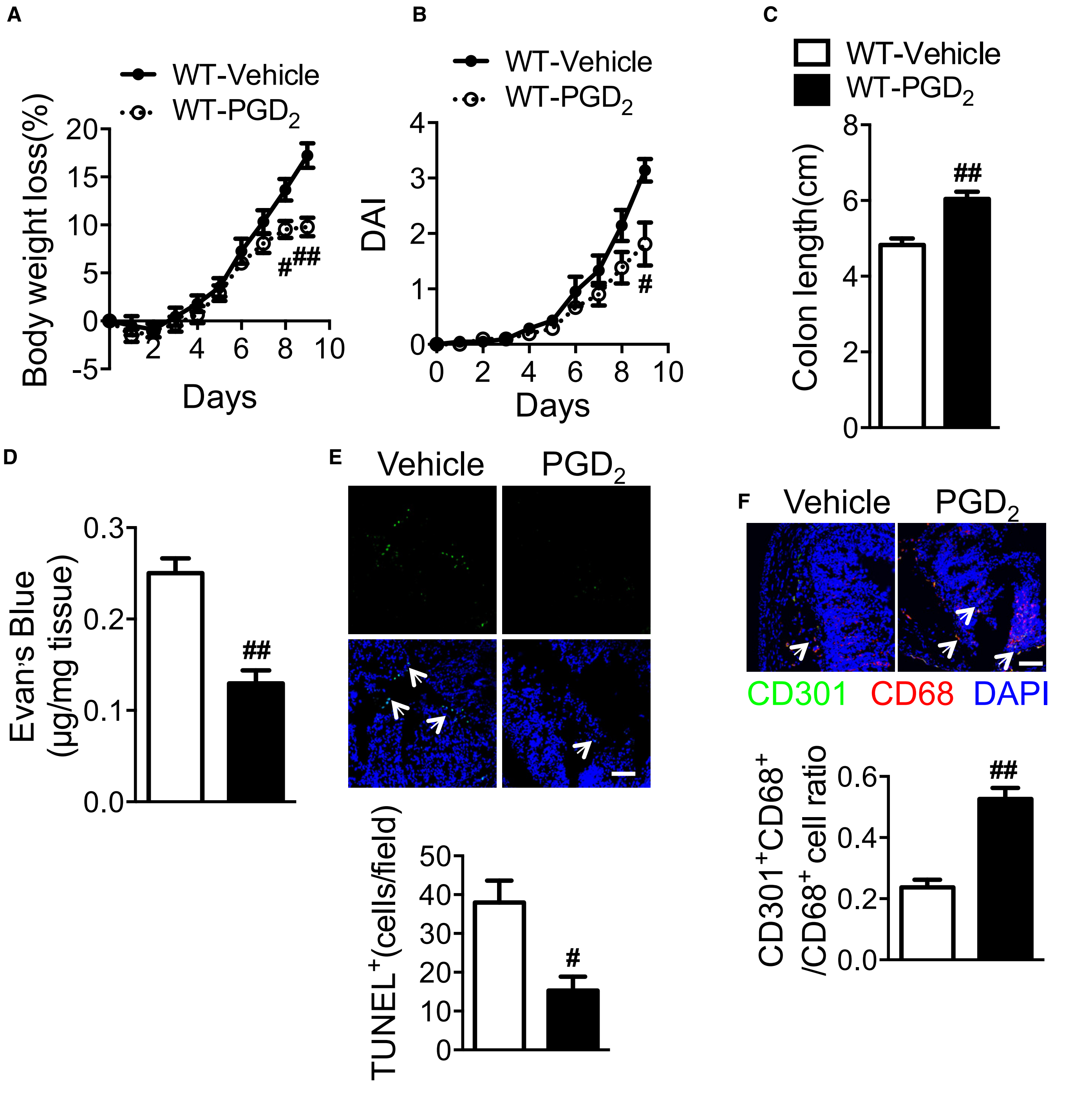

Figure EV5. PGD_2 infusion ameliorates DSS-induced colitis in mice.

A–C Effect of PGD_2 treatment on body weight loss (A), disease activity index (B), and colon length (C, centimeter) of WT mice in response to DSS challenge.
D Effect of PGD_2 on Evan's blue extravasation in the colonic mucosa from WT mice.
E Effect of PGD_2 on DSS-induced epithelial cell apoptosis in WT mice. Scale bars: 100 μm. The arrows indicate $TUNEL^+$ cells.
F Effect of PGD_2 on colonic macrophage infiltration in DSS-challenged WT mice. Scale bars: 100 μm. The arrows indicate $CD301^+CD68^+$ cells.

Data information: All mice were sacrificed at day 9. Data are shown as mean ± SEM. Statistical significance was determined using unpaired Student's *t*-tests. (A–C) $^{\#}P < 0.05$, $^{\#\#}P < 0.01$ vs. vehicle; $n = 7$. (D) $^{\#\#}P < 0.01$ compared with vehicle; $n = 7$. (E) $^{\#}P < 0.05$ compared with vehicle; vehicle, $n = 5$; PGD_2, $n = 7$. (F) $^{\#\#}P < 0.01$ compared with vehicle; $n = 5$.

© 2017 The Authors
